# 4-Hy­droxy-5-(2-meth­oxy­phen­oxy)-2,2′-bipyrimidin-6(5*H*)-one dihydrate

**DOI:** 10.1107/S1600536813028900

**Published:** 2013-10-26

**Authors:** Thammarse S. Yamuna, Jerry P. Jasinski, Brian J. Anderson, H.S. Yathirajan, Manpreet Kaur

**Affiliations:** a Department of Studies in Chemistry, University of Mysore, Manasagangotri, Mysore 570 006, India; b Department of Chemistry, Keene State College, 229 Main Street, Keene, NH 03435-2001, USA

## Abstract

The title compound, C_15_H_12_N_4_O_4_·2H_2_O, crystallizes with two independent water mol­ecules in the asymmetric unit. The dihedral angles between the mean planes of the benzene and pyrimidine rings and that of the pyrimidin-4-one ring are 85.1 (9) and 82.1 (1)°, respectively. The mean plane of the pyrimidine ring is twisted by 12.8 (8)° from that of the pyrimidin-4-one ring. The dihedral angles between the benzene ring and the mean planes of the pyrimidine and pyrimidin-4-one rings are 85.1 (9) and 82.1 (1)°, respectively.In the crystal, N–H⋯O, O—H⋯N and O—H⋯O hydrogen bonds involving both water mol­ecules are present; these link the mol­ecules into a two-dimensional network parallel to (010). In addition, weak C—H⋯π and π–π [centroid–centroid distance = 3.6183 (8) Å] inter­actions occur.

## Related literature

For substituted pyrimidine-2,4-diones as good reversible inhibitors of thymidine phospho­rylase, see: Baker & Rzeszotarki (1967 ▶). For the use of 2,2′-bi­pyrimidine as a ligand in inorganic and organometallic chemistry, see: Hunziker & Ludi (1977 ▶); Fabrice *et al.* (2008 ▶). For related structures, see: El-Brollosy *et al.* (2012 ▶); Fun *et al.* (2009 ▶); Kaur *et al.* (2013 ▶); Ren *et al.* (2011 ▶); Trilleras *et al.* (2009 ▶).
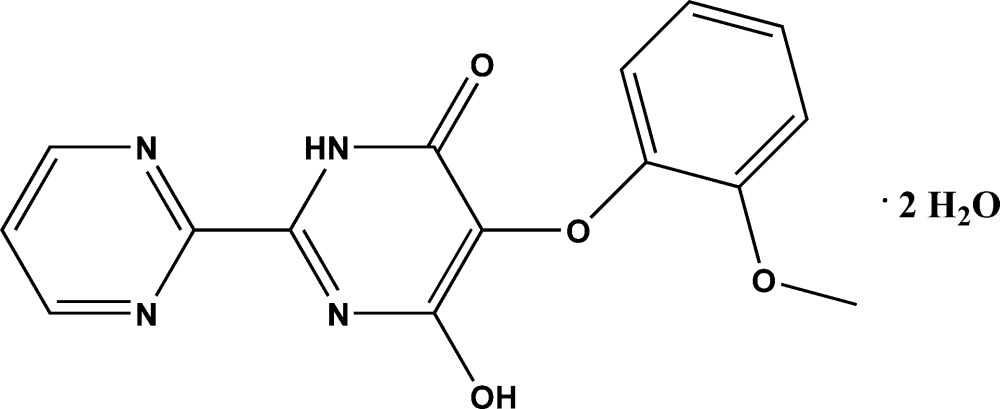


## Experimental

### 

#### Crystal data

C_15_H_12_N_4_O_4_·2H_2_O
*M* *_r_* = 348.32
Monoclinic, 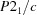

*a* = 9.5817 (3) Å
*b* = 13.9439 (3) Å
*c* = 12.4804 (4) Å
β = 109.832 (3)°
*V* = 1568.55 (8) Å^3^
*Z* = 4
Cu *K*α radiation
μ = 0.99 mm^−1^
*T* = 173 K
0.45 × 0.32 × 0.24 mm

#### Data collection

Agilent Xcalibur (Eos, Gemini) diffractometer
Absorption correction: multi-scan (*CrysAlis PRO* and *CrysAlis RED*; Agilent, 2012 ▶) *T*_min_ = 0.876, *T*_max_ = 1.000
9840 measured reflections
3070 independent reflections
2789 reflections with *I* > 2σ(*I*)
*R*_int_ = 0.040

#### Refinement

*R*[*F* ^2^ > 2σ(*F* ^2^)] = 0.041
*wR*(*F* ^2^) = 0.115
*S* = 1.03
3070 reflections
252 parameters
H atoms treated by a mixture of independent and constrained refinement
Δρ_max_ = 0.31 e Å^−3^
Δρ_min_ = −0.26 e Å^−3^

### 

Data collection: *CrysAlis PRO* (Agilent, 2012 ▶); cell refinement: *CrysAlis PRO*; data reduction: *CrysAlis RED* (Agilent, 2012 ▶); program(s) used to solve structure: *SUPERFLIP* (Palatinus & Chapuis, 2007 ▶); program(s) used to refine structure: *SHELXL2012* (Sheldrick, 2008 ▶); molecular graphics: *OLEX2* (Dolomanov *et al.*, 2009 ▶); software used to prepare material for publication: *OLEX2*.

## Supplementary Material

Crystal structure: contains datablock(s) I. DOI: 10.1107/S1600536813028900/bt6940sup1.cif

Structure factors: contains datablock(s) I. DOI: 10.1107/S1600536813028900/bt6940Isup2.hkl

Supplementary material file. DOI: 10.1107/S1600536813028900/bt6940Isup3.cml

Additional supplementary materials:  crystallographic information; 3D view; checkCIF report

## Figures and Tables

**Table 1:** Hydrogen-bond geometry (Å, °) *Cg*3 is the centroid of the C9–C14 ring.

*D*—H⋯*A*	*D*—H	H⋯*A*	*D*⋯*A*	*D*—H⋯*A*
O3—H3⋯O2*W* ^i^	0.91 (3)	1.67 (3)	2.5651 (15)	172 (2)
N4—H4⋯O1^ii^	0.88 (2)	2.08 (2)	2.9313 (15)	163.6 (18)
O1*W*—H1*WA*⋯O4^iii^	0.87 (2)	2.05 (2)	2.8917 (16)	163 (2)
O1*W*—H1*WB*⋯N2^iv^	0.89 (3)	2.01 (3)	2.8823 (16)	167 (2)
O2*W*—H2*WA*⋯O4	0.86 (2)	1.93 (3)	2.7803 (15)	172 (2)
O2*W*—H2*WB*⋯O1*W* ^v^	0.91 (3)	1.80 (3)	2.7050 (17)	173 (2)
C4—H4*A*⋯*Cg*3^vi^	0.95	2.82	3.4083 (16)	121

Symmetry codes: (i) 
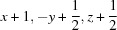
; (ii) 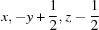; (iii) 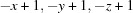; (iv) 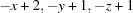; (v) 
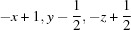
; (vi) 
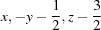
.

**Fig. 1.**
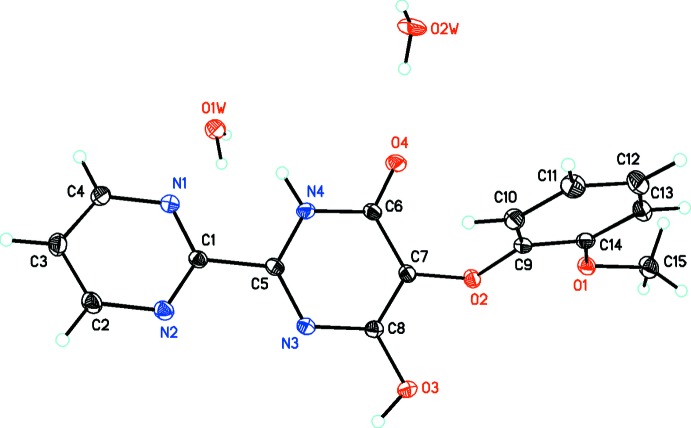
ORTEP drawing of (I) (C15H12N4O4. 2 H2O) showing the labeling scheme with 30% probability displacement ellipsoids.

**Fig. 2.**
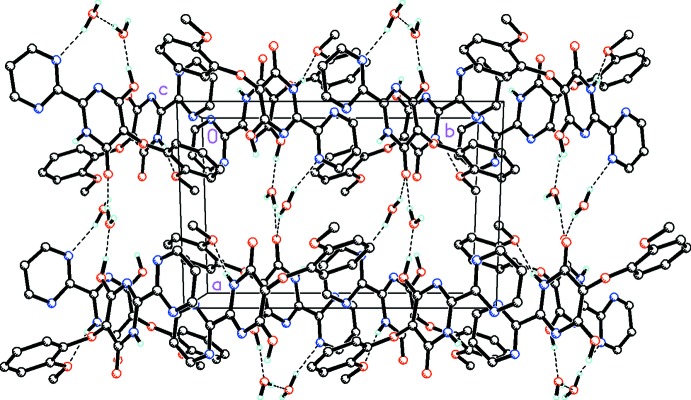
Molecular packing for (I) viewed along the C axis. Dashed lines indicate O—H···N and O—H···O hydrogen bonds involving both water molecules and N—H···O hydrogen bonds between the molecules. All of these interactions directly link the molecules into a 2D network along (0 1 0). H atoms not involved in hydrogen bonding have been removed for clarity.

## supplementary crystallographic information

### 1. Comment

The title compound, C_15_H_12_N_4_O_4_. 2 H_2_O, is an intermediate for the synthesis of bosentan. Pyrimidinediones are a class of chemical compounds characterized by a pyrimidine ring substituted with two carbonyl groups. A number of substituted pyrimidine-2,4-diones were synthesized and intensively studied as good reversible inhibitors of thymidine phosphorylase (Baker & Rzeszotarki,1967). 2,2'-Bipyrimidine has been used as a ligand in inorganic and organometallic chemistry (Hunziker & Ludi, 1977; Fabrice *et al.*, 2008). The crystal structures of some related compounds are: four 7-aryl-substituted pyrido[2,3-d]pyrimidine -2,4(1H,3H)-diones: similar molecular structures but different crystal structures (Trilleras *et al.*, 2009), diammonium 1,1',3,3'-tetra methyl-2,2',4,4',6,6'-hexaoxoperhydro-5,5'-bipyrimidine-5,5'-diide monohydrate (Fun *et al.*, 2009), 4,6-dichloro-5-(2-methoxy phenoxy)-2,2'-bipyrimidine (Ren *et al.*, 2011), 6-(3,5-dimethyl benzyl)-5-ethyl-1-[(3-phenylpropoxy) methyl]-1,2,3,4-tetrahydro pyrimidine-2,4-dione (El-Brollosy *et al.*, 2012), 4-tert-butyl- N-[6-(2-hydroxyethoxy)-5-(2-methoxyphenoxy)-2-(pyrimidin-2-yl)pyrimidin -4-yl]benzene-1-sulfonamide monohydrate (Kaur *et al.*, 2013), have been reported. In view of the importance of the title compound this paper reports its crystal structure.

The title compound crystallizes with two independent water molecules in the asymmetric unit (Fig. 1). In the molecule, the dihedral angle between the mean planes of the phenyl ring and pyrimidine ring and the pyrimidinyl-4-one is 85.1 (9)° and 82.1 (1)°, respectively. The mean plane of the pyrimidine ring is twisted by 12.8 (8)° from that of the pyrimidinyl-4-one ring. In addition, the hydroxy group and methoxy group are twisted from the pyrimidinyl-4-one and phenyl ring by -177.6 (8)° (H3/O3/C8/C7) and -173.3 (4)° (C9/C14/O1/C15), respectively. In the crystal, O—H···N and O—H···O hydrogen bonds involving both water molecules are present. N—H···O hydrogen bonds between molecules help strengthen the crystal lattice (Table. 1). In addition, weak C4—H4A···Cg3 and Cg1–Cg2 π–π intermolecular interactions are observed and contribute to crystal packing (Cg1–Cg2 = 3.6183 (8)Å; Cg1 = N1/C1/N2/C2/C3/C4; Cg2^i^ = N3/C5/N4/C6/C7/C8; symmetry operator (i) -x,1-y,1-z). The hydrogen bonds link the molecules into a 2D network parallel to (0 1 0) (Fig. 2).

### 2. Experimental

The title compound was obtained as a gift sample from Cadila Pharmaceuticals, Ahmedabad. The title compound (0.5 g) was dissolved in 10 ml of a mixture of dimethylsulphoxide and dimethylformamide (1:1) and stirred for 30 minutes on magnetic stirrer at 308 K. X-ray quality crystals of the title compound were formed after few days (m. p.: 536–540 K).

### 3. Refinement

H3, H4, H1WA, H1WB, H2WA and H2WB were located by a difference map and refined isotropically. All of the remaining H atoms were placed in their calculated positions and then refined using the riding model with Atom—H lengths of 0.95Å (CH) or 0.98Å (CH_3_). Isotropic displacement parameters for these atoms were set to 1.2 (CH) or 1.5 (CH_3_) times *U*_eq_ of the parent atom. The methyl group was refined as a rotating group.

### Crystal data

**Table:**

C_15_H_12_N_4_O_4_·2H_2_O	*F*(000) = 728
*M**_r_* = 348.32	*D*_x_ = 1.475 Mg m^−^^3^
Monoclinic, *P*2_1_/*c*	Cu *K*α radiation, λ = 1.54184 Å
*a* = 9.5817 (3) Å	Cell parameters from 4761 reflections
*b* = 13.9439 (3) Å	θ = 3.2–72.3°
*c* = 12.4804 (4) Å	µ = 0.99 mm^−^^1^
β = 109.832 (3)°	*T* = 173 K
*V* = 1568.55 (8) Å^3^	Irregular, colourless
*Z* = 4	0.45 × 0.32 × 0.24 mm

### Data collection

**Table:**

Agilent Xcalibur (Eos, Gemini) diffractometer	3070 independent reflections
Radiation source: Enhance (Cu) X-ray Source	2789 reflections with *I* > 2σ(*I*)
Detector resolution: 16.0416 pixels mm^-1^	*R*_int_ = 0.040
ω scans	θ_max_ = 72.5°, θ_min_ = 4.9°
Absorption correction: multi-scan (*CrysAlis PRO* and *CrysAlis RED*; Agilent, 2012)	*h* = −11→11
*T*_min_ = 0.876, *T*_max_ = 1.000	*k* = −17→13
9840 measured reflections	*l* = −15→14

### Refinement

**Table:**

Refinement on *F*^2^	Hydrogen site location: mixed
Least-squares matrix: full	H atoms treated by a mixture of independent and constrained refinement
*R*[*F*^2^ > 2σ(*F*^2^)] = 0.041	*w* = 1/[σ^2^(*F*_o_^2^) + (0.0697*P*)^2^ + 0.4106*P*] where *P* = (*F*_o_^2^ + 2*F*_c_^2^)/3
*wR*(*F*^2^) = 0.115	(Δ/σ)_max_ < 0.001
*S* = 1.03	Δρ_max_ = 0.31 e Å^−^^3^
3070 reflections	Δρ_min_ = −0.26 e Å^−^^3^
252 parameters	Extinction correction: *SHELXL2012* (Sheldrick, 2008), Fc^*^=kFc[1+0.001xFc^2^λ^3^/sin(2θ)]^-1/4^
0 restraints	Extinction coefficient: 0.0118 (8)
Primary atom site location: structure-invariant direct methods	

### Special details

**Table:**

Geometry. All esds (except the esd in the dihedral angle between two l.s. planes) are estimated using the full covariance matrix. The cell esds are taken into account individually in the estimation of esds in distances, angles and torsion angles; correlations between esds in cell parameters are only used when they are defined by crystal symmetry. An approximate (isotropic) treatment of cell esds is used for estimating esds involving l.s. planes.

### Fractional atomic coordinates and isotropic or equivalent isotropic displacement parameters (Å^2^)

**Table:**

	*x*	*y*	*z*	*U*_iso_*/*U*_eq_	
O1	0.66810 (10)	0.07758 (7)	0.72585 (8)	0.0234 (2)	
O2	0.83857 (10)	0.17796 (7)	0.64227 (8)	0.0218 (2)	
O3	1.14014 (11)	0.20971 (7)	0.71581 (8)	0.0261 (3)	
H3	1.235 (3)	0.2265 (17)	0.727 (2)	0.059 (7)*	
O4	0.67224 (10)	0.27899 (8)	0.44713 (8)	0.0284 (3)	
N1	0.99634 (13)	0.47988 (8)	0.33822 (10)	0.0233 (3)	
N2	1.24321 (12)	0.42848 (8)	0.43964 (10)	0.0228 (3)	
N3	1.12057 (12)	0.31273 (8)	0.56724 (9)	0.0199 (3)	
N4	0.88527 (12)	0.34237 (8)	0.43264 (10)	0.0214 (3)	
C1	1.09611 (14)	0.42507 (9)	0.41329 (11)	0.0195 (3)	
C2	1.29246 (15)	0.49577 (11)	0.38523 (12)	0.0268 (3)	
H2	1.3965	0.5022	0.4023	0.032*	
C3	1.19918 (17)	0.55649 (10)	0.30517 (12)	0.0284 (3)	
H3A	1.2366	0.6040	0.2675	0.034*	
C4	1.04900 (16)	0.54475 (10)	0.28272 (12)	0.0261 (3)	
H4A	0.9812	0.5838	0.2264	0.031*	
C5	1.03430 (14)	0.35477 (9)	0.47561 (11)	0.0189 (3)	
C6	0.80944 (14)	0.28493 (9)	0.48529 (11)	0.0205 (3)	
C7	0.90348 (14)	0.23650 (9)	0.58376 (11)	0.0196 (3)	
C8	1.05471 (14)	0.25246 (9)	0.62200 (11)	0.0198 (3)	
C9	0.79005 (14)	0.08924 (9)	0.59160 (11)	0.0202 (3)	
C10	0.82984 (16)	0.05252 (10)	0.50337 (12)	0.0255 (3)	
H10	0.8894	0.0895	0.4715	0.031*	
C11	0.78253 (18)	−0.03891 (11)	0.46092 (13)	0.0314 (3)	
H11	0.8091	−0.0640	0.3997	0.038*	
C12	0.69761 (19)	−0.09249 (11)	0.50761 (14)	0.0341 (4)	
H12	0.6664	−0.1550	0.4792	0.041*	
C13	0.65690 (17)	−0.05569 (11)	0.59662 (13)	0.0294 (3)	
H13	0.5980	−0.0931	0.6285	0.035*	
C14	0.70226 (14)	0.03557 (10)	0.63884 (11)	0.0211 (3)	
C15	0.59236 (18)	0.01817 (12)	0.78172 (13)	0.0329 (4)	
H15A	0.5822	0.0526	0.8472	0.049*	
H15B	0.6492	−0.0409	0.8080	0.049*	
H15C	0.4937	0.0021	0.7283	0.049*	
O1W	0.50931 (13)	0.68034 (8)	0.41460 (10)	0.0335 (3)	
O2W	0.41708 (12)	0.25759 (10)	0.25780 (10)	0.0385 (3)	
H2WA	0.493 (3)	0.2699 (16)	0.316 (2)	0.049 (6)*	
H2WB	0.445 (3)	0.2361 (16)	0.199 (2)	0.055 (6)*	
H4	0.836 (2)	0.3704 (14)	0.3675 (18)	0.037 (5)*	
H1WA	0.460 (3)	0.7048 (16)	0.455 (2)	0.050 (6)*	
H1WB	0.592 (3)	0.6557 (17)	0.464 (2)	0.058 (7)*	

### Atomic displacement parameters (Å^2^)

**Table:**

	*U*^11^	*U*^22^	*U*^33^	*U*^12^	*U*^13^	*U*^23^
O1	0.0213 (5)	0.0276 (5)	0.0228 (5)	−0.0045 (4)	0.0095 (4)	−0.0001 (4)
O2	0.0242 (5)	0.0226 (5)	0.0198 (5)	−0.0055 (3)	0.0091 (4)	−0.0022 (3)
O3	0.0201 (5)	0.0306 (5)	0.0234 (5)	−0.0006 (4)	0.0017 (4)	0.0072 (4)
O4	0.0149 (5)	0.0421 (6)	0.0260 (5)	−0.0032 (4)	0.0041 (4)	0.0052 (4)
N1	0.0211 (6)	0.0246 (6)	0.0225 (6)	−0.0014 (4)	0.0051 (4)	0.0012 (4)
N2	0.0186 (6)	0.0276 (6)	0.0228 (6)	−0.0015 (4)	0.0080 (4)	−0.0011 (4)
N3	0.0162 (5)	0.0217 (6)	0.0207 (6)	−0.0004 (4)	0.0049 (4)	−0.0004 (4)
N4	0.0160 (5)	0.0271 (6)	0.0195 (6)	0.0000 (4)	0.0036 (4)	0.0034 (4)
C1	0.0191 (6)	0.0214 (6)	0.0181 (6)	−0.0020 (5)	0.0062 (5)	−0.0034 (5)
C2	0.0213 (7)	0.0352 (8)	0.0266 (7)	−0.0055 (5)	0.0115 (6)	−0.0021 (6)
C3	0.0341 (8)	0.0287 (7)	0.0249 (7)	−0.0079 (6)	0.0135 (6)	−0.0001 (5)
C4	0.0294 (7)	0.0245 (7)	0.0220 (7)	−0.0022 (5)	0.0056 (5)	0.0015 (5)
C5	0.0163 (6)	0.0202 (6)	0.0204 (6)	−0.0004 (4)	0.0064 (5)	−0.0028 (5)
C6	0.0169 (6)	0.0250 (7)	0.0196 (6)	−0.0029 (5)	0.0061 (5)	−0.0025 (5)
C7	0.0199 (7)	0.0204 (6)	0.0193 (6)	−0.0031 (5)	0.0076 (5)	−0.0016 (5)
C8	0.0196 (6)	0.0197 (6)	0.0188 (6)	0.0010 (5)	0.0047 (5)	−0.0012 (5)
C9	0.0176 (6)	0.0214 (6)	0.0187 (6)	−0.0010 (5)	0.0022 (5)	0.0001 (5)
C10	0.0268 (7)	0.0274 (7)	0.0236 (7)	−0.0028 (5)	0.0101 (5)	−0.0012 (5)
C11	0.0385 (8)	0.0304 (8)	0.0259 (7)	−0.0003 (6)	0.0117 (6)	−0.0065 (6)
C12	0.0416 (9)	0.0246 (7)	0.0333 (8)	−0.0079 (6)	0.0090 (7)	−0.0081 (6)
C13	0.0302 (7)	0.0274 (7)	0.0297 (7)	−0.0087 (6)	0.0090 (6)	−0.0007 (6)
C14	0.0176 (6)	0.0258 (7)	0.0179 (6)	−0.0007 (5)	0.0034 (5)	0.0008 (5)
C15	0.0335 (8)	0.0399 (8)	0.0299 (8)	−0.0119 (6)	0.0167 (6)	0.0000 (6)
O1W	0.0237 (6)	0.0415 (6)	0.0361 (6)	0.0028 (4)	0.0114 (5)	0.0038 (5)
O2W	0.0189 (5)	0.0652 (8)	0.0275 (6)	−0.0042 (5)	0.0028 (5)	−0.0095 (5)

### Geometric parameters (Å, º)

**Table:**

O1—C14	1.3676 (16)	C4—H4A	0.9500
O1—C15	1.4292 (16)	C6—C7	1.4237 (19)
O2—C7	1.3765 (15)	C7—C8	1.3812 (18)
O2—C9	1.3952 (16)	C9—C10	1.3802 (19)
O3—H3	0.91 (3)	C9—C14	1.3970 (18)
O3—C8	1.3212 (16)	C10—H10	0.9500
O4—C6	1.2398 (16)	C10—C11	1.396 (2)
N1—C1	1.3297 (18)	C11—H11	0.9500
N1—C4	1.3378 (18)	C11—C12	1.372 (2)
N2—C1	1.3351 (17)	C12—H12	0.9500
N2—C2	1.3347 (18)	C12—C13	1.394 (2)
N3—C5	1.3013 (17)	C13—H13	0.9500
N3—C8	1.3653 (17)	C13—C14	1.390 (2)
N4—C5	1.3554 (16)	C15—H15A	0.9800
N4—C6	1.3874 (17)	C15—H15B	0.9800
N4—H4	0.88 (2)	C15—H15C	0.9800
C1—C5	1.4930 (17)	O1W—H1WA	0.87 (2)
C2—H2	0.9500	O1W—H1WB	0.89 (3)
C2—C3	1.381 (2)	O2W—H2WA	0.86 (2)
C3—H3A	0.9500	O2W—H2WB	0.91 (3)
C3—C4	1.379 (2)		
			
C14—O1—C15	115.91 (11)	O3—C8—N3	117.94 (12)
C7—O2—C9	115.24 (10)	O3—C8—C7	119.70 (12)
C8—O3—H3	108.2 (15)	N3—C8—C7	122.35 (12)
C1—N1—C4	116.50 (12)	O2—C9—C14	116.07 (11)
C2—N2—C1	115.19 (12)	C10—C9—O2	123.42 (12)
C5—N3—C8	116.97 (11)	C10—C9—C14	120.45 (12)
C5—N4—C6	122.48 (11)	C9—C10—H10	120.0
C5—N4—H4	118.2 (13)	C9—C10—C11	119.98 (13)
C6—N4—H4	119.3 (13)	C11—C10—H10	120.0
N1—C1—N2	126.84 (12)	C10—C11—H11	120.0
N1—C1—C5	115.28 (11)	C12—C11—C10	119.95 (13)
N2—C1—C5	117.86 (11)	C12—C11—H11	120.0
N2—C2—H2	118.5	C11—C12—H12	119.8
N2—C2—C3	123.04 (13)	C11—C12—C13	120.31 (14)
C3—C2—H2	118.5	C13—C12—H12	119.8
C2—C3—H3A	121.7	C12—C13—H13	119.9
C4—C3—C2	116.66 (13)	C14—C13—C12	120.22 (13)
C4—C3—H3A	121.7	C14—C13—H13	119.9
N1—C4—C3	121.72 (13)	O1—C14—C9	116.50 (12)
N1—C4—H4A	119.1	O1—C14—C13	124.41 (12)
C3—C4—H4A	119.1	C13—C14—C9	119.09 (12)
N3—C5—N4	124.06 (12)	O1—C15—H15A	109.5
N3—C5—C1	120.51 (11)	O1—C15—H15B	109.5
N4—C5—C1	115.37 (11)	O1—C15—H15C	109.5
O4—C6—N4	120.94 (12)	H15A—C15—H15B	109.5
O4—C6—C7	125.30 (12)	H15A—C15—H15C	109.5
N4—C6—C7	113.76 (11)	H15B—C15—H15C	109.5
O2—C7—C6	118.15 (11)	H1WA—O1W—H1WB	107 (2)
O2—C7—C8	121.47 (12)	H2WA—O2W—H2WB	111 (2)
C8—C7—C6	120.29 (12)		
			
O2—C7—C8—O3	1.07 (19)	C5—N4—C6—O4	176.19 (12)
O2—C7—C8—N3	−177.80 (11)	C5—N4—C6—C7	−3.53 (18)
O2—C9—C10—C11	176.86 (13)	C6—N4—C5—N3	2.0 (2)
O2—C9—C14—O1	2.85 (17)	C6—N4—C5—C1	−175.25 (11)
O2—C9—C14—C13	−176.50 (12)	C6—C7—C8—O3	177.58 (12)
O4—C6—C7—O2	0.1 (2)	C6—C7—C8—N3	−1.29 (19)
O4—C6—C7—C8	−176.55 (13)	C7—O2—C9—C10	13.23 (18)
N1—C1—C5—N3	−164.82 (12)	C7—O2—C9—C14	−169.66 (11)
N1—C1—C5—N4	12.53 (16)	C8—N3—C5—N4	0.20 (19)
N2—C1—C5—N3	13.60 (18)	C8—N3—C5—C1	177.32 (11)
N2—C1—C5—N4	−169.04 (11)	C9—O2—C7—C6	76.38 (14)
N2—C2—C3—C4	0.0 (2)	C9—O2—C7—C8	−107.04 (13)
N4—C6—C7—O2	179.77 (10)	C9—C10—C11—C12	−0.6 (2)
N4—C6—C7—C8	3.15 (18)	C10—C9—C14—O1	−179.95 (12)
C1—N1—C4—C3	−2.3 (2)	C10—C9—C14—C13	0.7 (2)
C1—N2—C2—C3	−1.5 (2)	C10—C11—C12—C13	0.7 (2)
C2—N2—C1—N1	1.32 (19)	C11—C12—C13—C14	−0.1 (2)
C2—N2—C1—C5	−176.90 (11)	C12—C13—C14—O1	−179.89 (13)
C2—C3—C4—N1	2.0 (2)	C12—C13—C14—C9	−0.6 (2)
C4—N1—C1—N2	0.5 (2)	C14—C9—C10—C11	−0.1 (2)
C4—N1—C1—C5	178.78 (11)	C15—O1—C14—C9	−173.34 (12)
C5—N3—C8—O3	−179.40 (11)	C15—O1—C14—C13	5.96 (19)
C5—N3—C8—C7	−0.51 (18)		

### Hydrogen-bond geometry (Å, º)

Cg3 is the centroid of the C9–C14 ring.

**Table:**

*D*—H···*A*	*D*—H	H···*A*	*D*···*A*	*D*—H···*A*
O3—H3···O2*W*^i^	0.91 (3)	1.67 (3)	2.5651 (15)	172 (2)
N4—H4···O1^ii^	0.88 (2)	2.08 (2)	2.9313 (15)	163.6 (18)
O1*W*—H1*WA*···O4^iii^	0.87 (2)	2.05 (2)	2.8917 (16)	163 (2)
O1*W*—H1*WB*···N2^iv^	0.89 (3)	2.01 (3)	2.8823 (16)	167 (2)
O2*W*—H2*WA*···O4	0.86 (2)	1.93 (3)	2.7803 (15)	172 (2)
O2*W*—H2*WB*···O1*W*^v^	0.91 (3)	1.80 (3)	2.7050 (17)	173 (2)
C4—H4*A*···*Cg*3^vi^	0.95	2.82	3.4083 (16)	121

Symmetry codes: (i) *x*+1, −*y*+1/2, *z*+1/2; (ii) *x*, −*y*+1/2, *z*−1/2; (iii) −*x*+1, −*y*+1, −*z*+1; (iv) −*x*+2, −*y*+1, −*z*+1; (v) −*x*+1, *y*−1/2, −*z*+1/2; (vi) *x*, −*y*−1/2, *z*−3/2.
